# Detection of vitellogenin incorporation into zebrafish oocytes by FITC fluorescence

**DOI:** 10.1186/1477-7827-9-45

**Published:** 2011-04-11

**Authors:** Yohei Matsuda, Yoshie Ito, Hisashi Hashimoto, Hayato Yokoi, Tohru Suzuki

**Affiliations:** 1 Laboratory of Marine Life Science and Genetics, Graduate School of Agricultural Science, Tohoku University, Aoba-ku, Sendai 981-8555, Japan; 2 Bioscience and Biotechnology Center, Nagoya University, Nagoya 464-8601, Japan; 3 Industrial Technology Institute, Miyagi Prefectural Government, Sendai, 981-3206, Japan

## Abstract

**Background:**

Large volumes of lymph can be collected from the eye-sacs of bubble-eye goldfish. We attempted to induce vitellogenin (Vtg) in the eye-sac lymph of bubble-eye goldfish and develop a method for visualizing Vtg incorporation by zebrafish oocytes using FITC-labeling.

**Methods:**

Estrogen efficiently induced Vtg in the eye-sac lymph of goldfish. After FITC-labeled Vtg was prepared, it was injected into mature female zebrafish.

**Results:**

Incorporation of FITC-labeled Vtg by zebrafish oocytes was detected in *in vivo *and *in vitro *experiments. The embryos obtained from zebrafish females injected with FITC-labeled Vtg emitted FITC fluorescence from the yolk sac and developed normally.

**Conclusion:**

This method for achieving Vtg incorporation by zebrafish oocytes could be useful in experiments related to the development and endocrinology of zebrafish oocytes.

## Background

Zebrafish (*Danio rerio*), a small teleost fish belonging to the family Cyprinidae, is widely used as a model organism in developmental biology and endocrinology. Most studies on oocyte development in zebrafish have focused on the elucidation of the molecular basis of vitellogenesis and oocyte maturation, with a particular emphasis on the molecular aspects of maternal mRNA accumulation associated with axial formation during early embryogenesis [1,2]. Vitellogenin (Vtg) is a female-specific phospholipoglycoprotein that is secreted by the liver and taken up by growing/vitellogenic oocytes where it is processed and stored as yolk proteins in the ooplasm [1,2]. The development of systems for both visualizing the incorporation of Vtg into oocytes and for allowing Vtg incorporation *in vitro *would be particularly useful in the fields of endocrinology and embryology. The *in vitro *oocyte culture systems that have been developed in the rainbow trout and eel to date both use Vtg prepared from the plasma of these species [3,4]. However, since obtaining sufficiently large volumes of zebrafish plasma for Vtg preparation is complicated by their small body size, developing an alternate source of Vtg for incorporation into the cytoplasm of zebrafish oocytes would be beneficial.

A system for synthesizing and incorporating Vtg into the oocytes of teleosts such as zebrafish has recently been reviewed [1,5]. Briefly, follicular stimulating hormone (FSH) from the pituitary stimulates the follicular cells surrounding the oocytes to produce estrogen (E2), which then stimulates Vtg synthesis in the liver. Once secreted into the plasma by the liver, the Vtg is quickly incorporated into the yolk granules of the oocytes by a plasma membrane Vtg-receptor (VtgR) system. The incorporation of Vtg into oocytes has been reported to be stimulated by FSH in rainbow trout (*Salmo gairdneri*) [6]. At this vitellogenic stage, the oocytes increase rapidly in size and accumulate fat-soluble vitamin A metabolites, such as retinal, which are essential for embryogenesis [7,8]. At the same time, maternal mRNAs related to germline specification and axial formation in embryos are transcribed and accumulate in the oocytes during vitellogenesis [2,9-11]. The Vtg that is stored in the yolk functions as a source of amino acids, lipids, and sugars during embryogenesis [1,2]. Interestingly, the administration of exogenous E2 has been experimentally shown to induce Vtg synthesis in the livers of both male and female rainbow trout [12].

**Figure 1:**
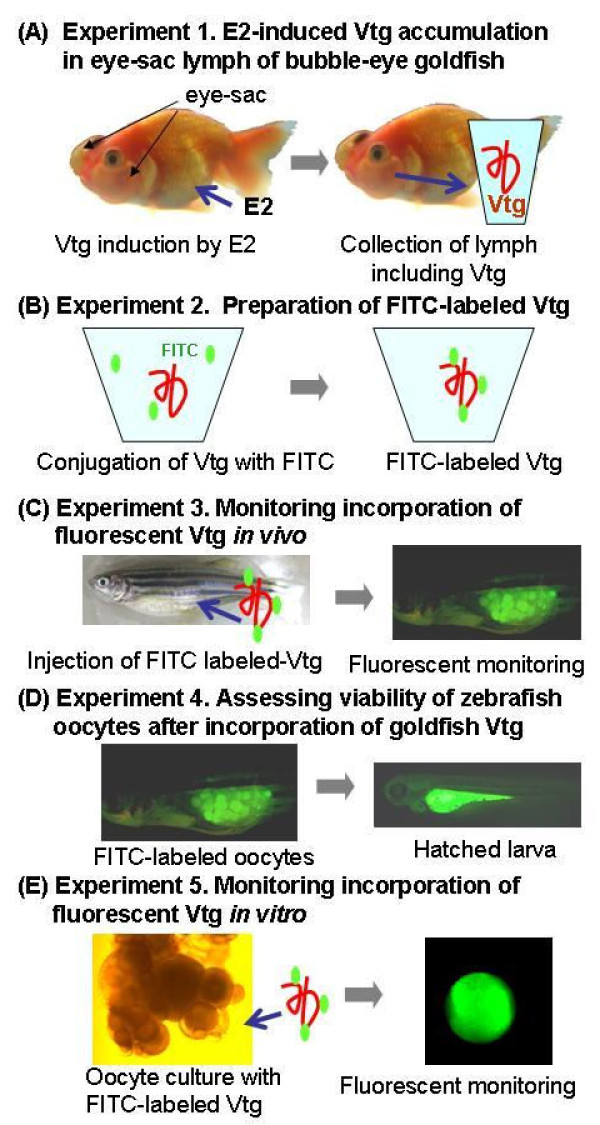
**Schematic representation of a system for monitoring Vtg incorporation into zebrafish oocytes using FITC-labeled Vtg prepared from bubble-eye goldfish**.

Bubble-eye goldfish, a variety of crucian carp (*Carassius auratus*) belonging to the same family as the zebrafish (Cyprinidae), are commonly sold at pet shops. The variety is characterized by having unique, sac-like structures (eye sacs) containing lymph below the eyes (Figure 1A). Relatively large quantities of this lymph can be collected with ease using a syringe. Since the eye-sac lymph does not clot after collection, it has been used to supplement the tissue culture media to stimulate the proliferation of zebrafish cells without any chemical or physical treatment after collection [13]. In addition, with 77.6% identity and 87.2% similarity, zebrafish Vtg1 (NP_001038362) and goldfish Vtg (ABG22139) are highly homologous with respect to their amino acid sequences. We therefore had the idea to induce Vtg in the lymph of the eye sacs of this goldfish by E2 administration and to use the Vtg harvested in this way as a source for experiments with zebrafish oocytes. We also established a system for visualizing the incorporation of Vtg into zebrafish oocytes by FITC labeling in the five experiments shown in Figure 1. Briefly, the first experiment examined whether Vtg could be induced in the eye-sac lymph of bubble-eye goldfish by E2 injection (Figure 1A). The second experiment attempted to label goldfish Vtg with FITC (Figure 1B). The third experiment examined whether zebrafish oocytes incorporated goldfish Vtg into the cytoplasm, using FITC fluorescence to trace the Vtg (Figure 1C). The fourth experiment assessed whether the zebrafish oocytes that accumulated exogenous FITC-labeled Vtg remained viable and whether embryogenesis could proceed normally (Figure 1D). The fifth experiment examined whether zebrafish oocytes incorporate FITC-labeled Vtg *in vitro *(Figure 1E).

## Methods

### Experiment 1: E2-induced Vtg accumulation in eye-sac lymph of bubble-eye goldfish

Bubble-eye goldfish and zebrafish were purchased from a local pet shop in Sendai, Japan. All of the fish were maintained at 28°C under photoperiod conditions of 14 h light and 10 h dark. Fish were anesthetized with MS-222 (Wako Pure Chemical Industries, Osaka, Japan) before E2 (Wako) injection and lymph collection. Fifty μl of dimethyl sulfoxide (Wako) containing E2 at 0.12 ng/μl was injected into the abdominal cavities of male and female bubble-eye goldfish weighing approximately 20 g. Lymph was collected from the eye-sac using a plastic syringe immediately before the injection of E2 and then 7 days thereafter. Collected lymph was boiled for 5 min with sodium dodecyl sulfate (SDS) and 2-mercaptoethanol (Wako), and then subjected to SDS-polyacrylamide gel electrophoresis (PAGE) [14]. The protein bands were stained with Coomassie Brilliant Blue R-250.

### Experiment 2: Preparation of FITC-labeled Vtg

For the preparation of FITC-labeled Vtg, E2 was injected into the abdominal cavity three times over a seven-day period to induce high concentrations of Vtg in eye-sac lymph. Lymph was then collected from the eye sac four days after the final E2 injection. A total of approximately 2 ml of lymph was obtained from both eye sacs of one fish. Obtained lymph was dialyzed against 0.05 M Na_2_CO_3_/NaHCO_3 _(sodium carbonate) buffer (pH9.5) overnight at 4°C. Fluorescein 5(6)-isothiocyanate (FITC; Wako) was dissolved in sodium carbonate buffer to a concentration of 10% and added to the dialyzed lymph at a ratio of 1:100, before being incubated at 4°C in the dark for 6 h to facilitate FITC conjugation. The lymph was then dialyzed twice against phosphate buffered saline (PBS) for 12 h at 4°C to remove unconjugated FITC; this solution is referred to as FITC-labeled Vtg solution hereafter. To confirm that FITC had covalently bonded to the Vtg, the sample was subjected to SDS-PAGE and irradiated with UV light to detect FITC-labeled proteins. As controls, we prepared FITC-labeled lymph proteins from male goldfish without E2 treatment and FITC-labeled bovine serum albumin (BSA; Rockland, Philadelphia, USA).

To separate the FITC-labeled Vtg from low molecular weight lymph proteins, 200 μl of FITC-labeled Vtg solution was eluted through a Sephacryl S-200 HR (GE Healthcare, Little Charfont, England) column (1 cm × 30 cm). Protein elution was monitored by FITC-fluorescence and the first peak was identified as the FITC-labeled, purified Vtg.

### Experiment 3: Monitoring incorporation of fluorescent Vtg *in vivo*

One hundred μl of FITC-labeled Vtg solution prepared as described above was injected into the abdominal cavities of zebrafish (n = 10) that had started spawning. Observations in zebrafish were performed after anesthetization with MS-222 and dissection of the left side of the abdomen. Incorporation of FITC-labeled Vtg into oocytes was examined using a Leica MZ15F fluorescence stereomicroscope (Wetzlar, Germany). Photographs were taken using a Leica DFC500 digital CCD camera (Wetzlar, Germany) and processed using Adobe Photoshop (version 7, Adobe Inc., San Jose, CA). To observe the intracellular localization of FITC-labeled Vtg, some of experimental fish were fixed in 4% paraformaldehyde in PBS, embedded in paraffin, and cut into 8 μm sections for observation under the fluorescence stereomicroscope after paraffin removal.

### Experiment 4: Assessing viability of zebrafish oocytes after incorporation of goldfish Vtg

Zebrafish females injected with FITC-labeled Vtg were maintained in a tank and mated with males. Fertilized eggs exhibiting FITC fluorescence were collected and reared.

### Experiment 5: Monitoring incorporation of fluorescent Vtg *in vitro*

The ovaries of a mature female zebrafish that had been anesthetized and sterilized in 0.2% sodium hypochlorite solution for 5 min were removed and washed three times in L15 medium. The ovaries were then separated into tissue explants containing 20-40 oocytes using forceps. Explants were then cultured at 28°C in the L15 medium as described by Sawatari et al. [13], except that 2% FITC labeled-Vtg solution, 5% fetal bovine serum (Sigma, St. Louis, MO), 200 ng/ml of carp pituitary acetone power (Sigma), and 5 mM N-2-hydroxyethylpiperazine-N'-2-ethanesulfonic acid (HEPES; Wako) (pH7.2) were added to the medium.

## Results

### Experiment 1

**Figure 2:**
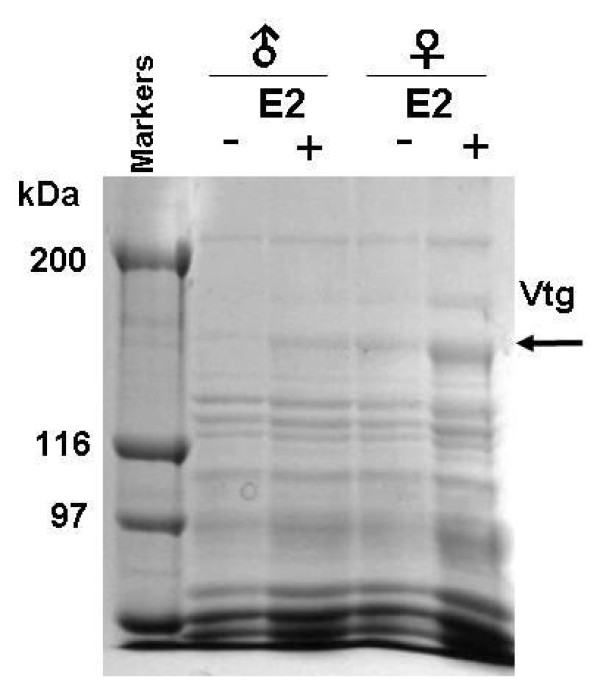
**Vtg induction in the eye sac of bubble-eye goldfish by E2 injection**. Lymph was collected from the eye sacs of male and female goldfish before (-) and after (+) E2 injection, and analyzed by SDS-PAGE. Position of the Vtg band is indicated by the arrow.

To determine whether Vtg could be induced in the eye-sac lymph by E2, the protein components of the lymph were compared before and after E2 injection by SDS-PAGE. Before stimulation by E2, a protein band of approximately 150 kDa was detected in female goldfish (Figure 2). Since biochemical assays have revealed that the molecular mass of Vtg from carp, *Cyprinus carpio*, which is phylogenetically closely related to goldfish, is also 150 kDa [15], the 150 kDa band in goldfish lymph was considered to be goldfish Vtg. At 7 days after E2 injection, the Vtg band was further enhanced in female lymph and newly induced in male lymph (Figure 2). It was thus shown that Vtg is inducible in the eye-sac lymph of both sexes of bubble-eye goldfish.

### Experiment 2

**Figure 3:**
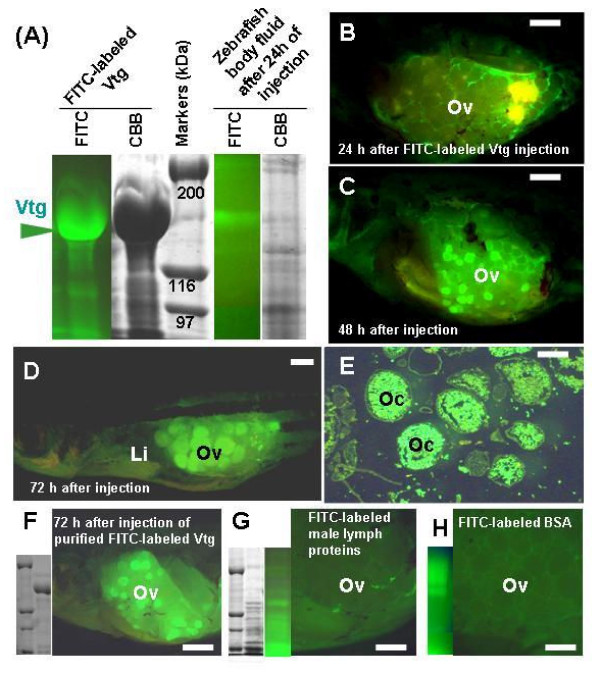
**Preparation and subsequent monitoring of FITC-labeled Vtg accumulation in the ovary after abdominal injection**. (A) SDS-PAGE analysis of eye-sac lymph proteins after FITC labeling (two leftmost lanes), and assessment of FITC-labeled Vtg stability in zebrafish body fluid (two rightmost lanes). Leftmost lane shows FITC-fluorescence, indicating strong emission from Vtg, and the next lane shows the same gel stained with Coomassie Brilliant Blue (CBB). Of the two rightmost lanes, the first shows FITC-fluorescence in zebrafish body fluid collected 24 h after injection of FITC-labeled Vtg solution into the abdominal cavity, and the next lane shows the same sample stained with CBB. (B-D) Fluorescent images of zebrafish with the left abdominal wall dissected at 1, 2 and 3 days after injection of FITC-labeled Vtg solution. (E) Section of the zebrafish ovary shown in D. (F) SDS-PAGE of purified FITC-labeled Vtg and its incorporation into oocytes. (G) SDS-PAGE of FITC-labeled male goldfish lymph, and zebrafish ovary 72 h after injection of this solution. (H) SDS-PAGE of FITC-labeled BSA, and zebrafish ovary 72 h after injection of this solution. Li: liver; Oc: oocyte; Ov: ovary. Scale bar = 2 mm in B-D and F-H, 200 μm in E.

We found that the three E2 injections administered over a seven-day period were sufficient for inducing Vtg as the predominant protein component of eye-sac lymph (Figure 3A). The lymph obtained after three E2 injections was conjugated with FITC in alkaline buffer as described in the Methods section above. After being processed with FITC, strong FITC-fluorescence emission was detected from the Vtg band by SDS-PAGE analysis (Figure 3A). Using this simple alkaline labeling method, FITC was considered to bind covalently to Vtg, and the FITC-labeled Vtg was a major fluorescent protein component of the prepared solution.

### Experiment 3

To examine whether heterogeneous Vtg prepared from goldfish could be incorporated by zebrafish oocytes, FITC-labeled Vtg solution prepared as described above was injected into the abdominal cavities of females and FITC fluorescence was then traced in the specimen under a fluorescent microscope. Although FITC fluorescence was detected in the ovary at 24 h after injection, incorporation into oocytes was not yet apparent (Figure 3B). However, at 48 and 72 h after injection, strong FITC fluorescence was detected in vitellogenic oocytes (Figure 3C,D), with sectioning of the ovaries revealing that yolk granules emitted FITC fluorescence (Figure 3E). When purified FITC-labeled Vtg was injected into females, zebrafish oocytes also started emitting FITC fluorescence (Figure 3F).

To examine the stability of goldfish Vtg in zebrafish, body fluid (approximately 1 μl) emitting FITC fluorescence was collected from the abdominal cavity of zebrafish under a fluorescence stereomicroscope at 24 h after injection, and subjected to SDS-PAGE analysis. A FITC-positive band was detected at the position of the original Vtg molecular weight, indicating that goldfish Vtg remained relatively stable in the zebrafish body fluid and did not degrade rapidly (Figure 3A).

As controls for determining whether zebrafish oocytes specifically incorporate goldfish Vtg as a yolk protein precursor, we prepared FITC-labeled, male lymph proteins from gold fish and FITC-labeled BSA (Figure 3G,H), and injected these into female zebrafish; neither of these control preparations was incorporated into in the oocytes (Figure 3G,H). Taken together, these findings indicated that the zebrafish oocytes incorporated the FITC-labeled goldfish Vtg into the yolk granules.

### Experiment 4

**Figure 4:**
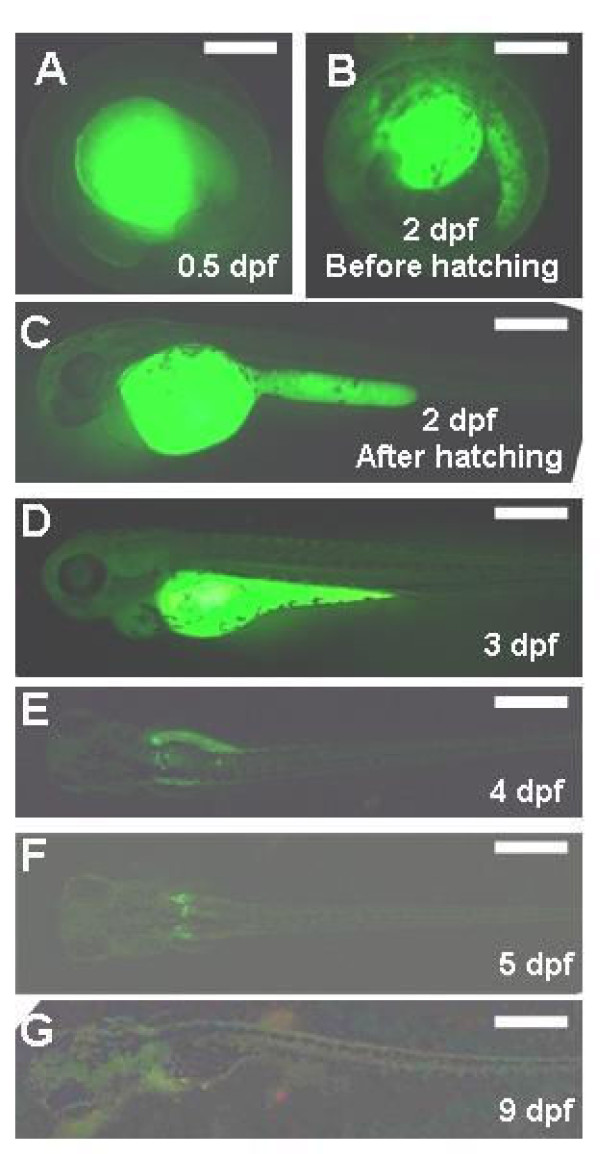
**Development of zebrafish embryos that incorporated FITC-labeled Vtg, heterogeneously prepared from the bubble-eye goldfish, in the yolk during oocyte growth**. Embryonic stage is given as days post fertilization (dpf). Scale bar = 200 μm.

We then examined the viability of zebrafish oocytes that had accumulated heterogeneous goldfish Vtg. Eight days after injection of the FITC-labeled Vtg into the abdominal cavity of mature females, eggs with yolks emitting FITC fluorescence were spawned by these females and were normally fertilized. Approximately 30 eggs were collected from each female and labeled with FITC. Compared with wild type embryos, the embryogenesis of these eggs was normal and all of the eggs hatched at 2 days post-fertilization (dpf) (Figure 4A-C). After hatching, the intensity of FITC fluorescence decreased rapidly as the yolk decreased in size, disappearing entirely by 9 dpf (Figure 4D-G). The results clearly showed that FITC-labeled Vtg prepared from the bubble-eye goldfish does not adversely affect zebrafish oocyte development and subsequent embryogenesis after being incorporated into the yolk.

### Experiment 5

**Figure 5:**
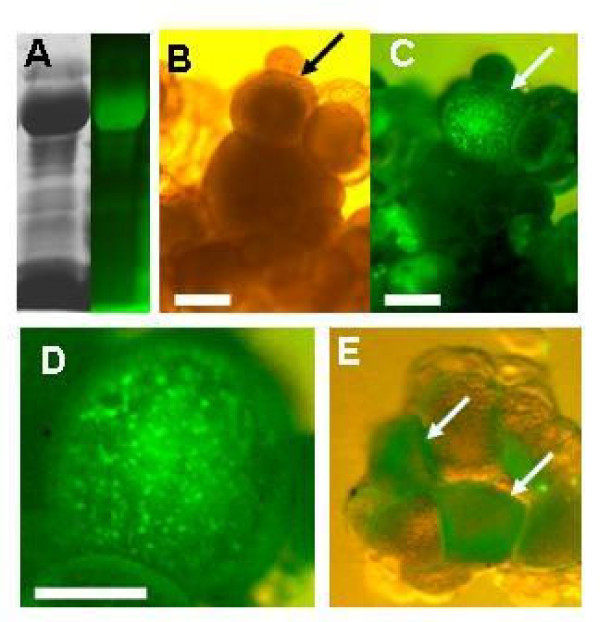
***In vitro *incorporation of FITC-labeled Vtg derived from the bubble-eye goldfish by zebrafish oocytes**. (A) Stability of FITC-labeled Vtg in the culture medium. The medium was subjected to SDS-PAGE after 48 h of incubation at 28°C. (B) Ovarian explant at 48 h of culture with FITC-labeled Vtg solution. (C) FITC fluorescence in the explant. (D) Enlargement of the ovary emitting FITC fluorescence. (E) Ovary explant cultured with purified FITC-labeled Vtg. Arrows show ovaries emitting FITC fluorescence. Scale bar = 200 μm.

Finally, we sought to determine whether FITC-labeled Vtg prepared from goldfish is incorporated into zebrafish oocytes under cell culture conditions. Dissected ovary explants were cultured using standard culture conditions [13] with the addition of 2% FITC-labeled Vtg solution. During culture for 48 h, goldfish Vtg was not markedly degraded in the culture medium containing fetal bovine serum (Figure 5A). At 48 h, florescence was detected in some of the vitellogenic oocytes in the ovary explants cultured with FITC-labeled Vtg solution (Figure 5B,C). Oocytes emitted granular fluorescence, indicating incorporation of FITC-labeled Vtg into yolk granules (Figure 5D). Purified FITC-labeled Vtg, shown in Figure 3F, was also incorporated by some of the ovaries under culture conditions (Figure 5E).

## Discussion

We demonstrated that Vtg can be induced by E2 injection in the eye-sac lymph of bubble-eye goldfish, and that the induced Vtg can be conjugated with FITC using a standard technique. Both female and male goldfish can be used for Vtg preparation, with three injections of E2 administered over a seven-day period being sufficient for obtaining enough Vtg for FITC-labeling. We also demonstrated that it was possible to monitor Vtg incorporation into zebrafish oocytes by FITC fluorescence, both *in vivo *and *in vitro*. By using a recently developed transparent zebrafish line [16], it would be possible to monitor the incorporation of FITC-labeled Vtg in a living specimen.

In addition, when accumulated in yolk, goldfish Vtg did not affect zebrafish oocyte development and subsequent embryogenesis, even though the Vtg was heterogeneous in origin. Approximately 2 ml of lymph can be harvested from the eye-sacs of bubble-eye goldfish weighing 20 g, which is sufficient for performing the Vtg incorporation experiment in 20 zebrafish and for preparing 60 ml of culture medium for examining Vtg incorporation into oocytes. The eye-sac lymph of bubble-eye goldfish is thus considered to be a superior source of Vtg for developmental and endocrine experiments using zebrafish oocytes.

This method is important because there are two possible supplemental sources of exogenous Vtg for oocyte development, recombinant Vtg and plasma- or lymph-derived Vtg. Since preparing a structurally complicated phospholipoglycoprotein, like Vtg, using recombinant protein methods is difficult, the application of plasma- or lymph-derived Vtg is considered to be preferable. We propose that Vtg induced in the eye-sac lymph is well suited for use as a source of Vtg for studies on zebrafish oocyte development. In addition, several possible applications of FITC-labeled Vtg prepared from goldfish exist. First, the effects of hormones on Vtg incorporation into zebrafish oocytes could be quantified by determining the FITC content of oocytes after hormonal treatment. Second, further development of a culture system using FITC-labeled Vtg may facilitate the culture of zebrafish oocytes *in vitro*. Third, goldfish Vtg may function as a carrier for introducing external proteins, such as antibodies, into zebrafish oocytes by conjugation with Vtg.

## Conclusion

We developed methods for preparing FITC-labeled Vtg from the eye-sac lymph of bubble-eye goldfish and for visualizing Vtg incorporation by zebrafish oocytes in *in vitro *and *in vivo *systems using FITC-labeled goldfish Vtg.

## Competing interests

The authors declare that they have no competing interests.

## Authors' contributions

YM, YI and TS performed the experiments. HS and HY interpreted the data and participated in the writing of the manuscript. TS conceived and designed the study and wrote the manuscript. All authors have read and approved the final manuscript.
